# The effect of smartphone-based monitoring and treatment including clinical feedback versus smartphone-based monitoring without clinical feedback in bipolar disorder: the SmartBipolar trial—a study protocol for a randomized controlled parallel-group trial

**DOI:** 10.1186/s13063-023-07625-1

**Published:** 2023-09-12

**Authors:** Maria Faurholt-Jepsen, Natacha Blauenfeldt Kyster, Malene Schwarz Dyreholt, Ellen Margrethe Christensen, Pernille Bondo-Kozuch, Anna Skovgaard Lerche, Birte Smidt, Ulla Knorr, Kim Brøndmark, Anne-Marie Bangsgaard Cardoso, Anja Mathiesen, Rene Sjælland, Henrik Nørbak-Emig, Lotte Linnemann Sponsor, Darius Mardosas, Ida Palmblad Sarauw-Nielsen, Jens Drachmann Bukh, Trine Vøgg Heller, Mads Frost, Nanna Iversen, Jakob Eyvind Bardram, Jonas Busk, Maj Vinberg, Lars Vedel Kessing

**Affiliations:** 1 Copenhagen Affective Disorder Research Center (CADIC), Psychiatric Center Copenhagen, Hovedvejen 17, 1. Floor, 2000 Frederiksberg, Denmark; 2 Department of Clinical Medicine, Faculty of Health and Medical Sciences, University of Copenhagen, Copenhagen, Denmark; 3 The Early Multimodular Prevention and Intervention Research Institution (EMPIRI), Mental Health Centre, Northern Zealand, Copenhagen University Hospital – Mental Health Services CPH, Copenhagen, Denmark; 4 Psychiatric Center Amager, Copenhagen, Denmark; 5 Psychiatric Center Glostrup, Glostrup, Denmark; 6 Psychiatric Center Ballerup, Ballerup, Denmark; 7 Monsenso A/S, Copenhagen, Denmark; 8 Department of Health Technology, Technical University of Denmark, Lyngby, Denmark; 9 Department of Energy Conversion and Storage, Technical University of Denmark, Lyngby, Denmark

**Keywords:** Bipolar disorder, Smartphone-based monitoring, Randomized controlled trial

## Abstract

**Introduction:**

A substantial proportion of patients with bipolar disorder experience daily subsyndromal mood swings, and the term “mood instability” reflecting the variability in mood seems associated with poor prognostic factors, including impaired functioning, and increased risk of hospitalization and relapse.

During the last decade, we have developed and tested a smartphone-based system for monitoring bipolar disorder. The present SmartBipolar randomized controlled trial (RCT) aims to investigate whether (1) daily smartphone-based outpatient monitoring and treatment *including* clinical feedback versus (2) daily smartphone-based monitoring *without* clinical feedback or (3) daily smartphone-based mood monitoring *only* improves mood instability and other clinically relevant patient-related outcomes in patients with bipolar disorder.

**Methods and analysis:**

The SmartBipolar trial is a pragmatic randomized controlled parallel-group trial. Patients with bipolar disorder are invited to participate as part of their specialized outpatient treatment for patients with bipolar disorder in Mental Health Services in the Capital Region of Denmark. The included patients will be randomized to (1) daily smartphone-based monitoring and treatment *including* a clinical feedback loop (intervention group) or (2) daily smartphone-based monitoring *without* a clinical feedback loop (control group) or (3) daily smartphone-based mood monitoring only (control group). All patients receive specialized outpatient treatment for bipolar disorder in the Mental Health Services in the Capital Region of Denmark. The trial started in March 2021 and has currently included 150 patients. The outcomes are (1) mood instability (primary), (2) quality of life, self-rated depressive symptoms, self-rated manic symptoms, perceived stress, satisfaction with care, cumulated number and duration of psychiatric hospitalizations, and medication (secondary), and (3) smartphone-based measures per month of stress, anxiety, irritability, activity, and sleep as well as the percentage of days with presence of mixed mood, days with adherence to medication and adherence to smartphone-based self-monitoring. A total of 201 patients with bipolar disorder will be included in the SmartBipolar trial.

**Ethics and dissemination:**

The SmartBipolar trial is funded by the Capital Region of Denmark and the Independent Research Fund Denmark. Ethical approval has been obtained from the Regional Ethical Committee in The Capital Region of Denmark (H-19067248) as well as data permission (journal number: P-2019–809). The results will be published in peer-reviewed academic journals, presented at scientific meetings, and disseminated to patients’ organizations and media outlets.

**Trial registration:**

Trial registration number: NCT04230421. Date March 1, 2021. Version 1.

**Supplementary Information:**

The online version contains supplementary material available at 10.1186/s13063-023-07625-1.

## Introduction

Bipolar disorder is characterized by recurrent alterations in mood, encompassing affective episodes of depression, (hypo)mania, and mixed episodes with intervening periods of euthymia [1]. Bipolar disorder is a disabling psychiatric disorder with a prevalence of 1–2%, a high risk of recurrence of affective episodes [2], a lifelong elevated risk of suicide, and a decreased life expectance of 8–12 years compared to the general population [3]. Bipolar disorder is a complex illness as patients present differently during manic, depressed, mixed, and remitted states, and also the treatment differs during these states. Despite current standard treatment, patients with bipolar disorder experience affective symptoms approximately half of the time, alternating between depressive episodes and manic episodes three times per year on average, while 10% suffer from permanent symptoms [4]. During the last decade, there has been a gradual shift from focusing on affective episodes to inter-episodic mood instability [5–8]. A substantial proportion of patients with bipolar disorder experience subsyndromal mood swings on a daily basis more than 50% of the time between episodes [8–10]. This mood variation (variability in mood registrations) is reflected as mood instability reflecting core psychopathology in bipolar disorder [9–12]. Furthermore, mood instability has been associated with increased perceived stress, decreased quality of life and functioning, and increased risk of relapse and hospitalization [9, 12–15]. In addition, mood instability has been suggested to be associated with biological measures of stress-hormone dynamics [16]. Due to the increased focus on the impact of mood instability, mood instability has been suggested as an independent treatment target and as a more sensitive outcome measure in randomized controlled trials (RCTs) than for example relapse or recurrence of affective episodes [7, 8, 11, 17, 18].

### A new organization for all patients with bipolar disorder: the Clinical Academic Group for bipolar disorder (the CAG Bipolar) in the Mental Health Services, Capital Region of Denmark

The present research group has initiated and conducted the first large pragmatic randomized controlled trial (RCT) covering the entire Mental Health Services, Capital Region of Denmark, in patients with newly diagnosed bipolar disorder investigating differences in effectiveness between specialized outpatient treatment and generalist treatment. The trial showed that *specialized* combined optimized pharmacological treatment, and group-based psychoeducation improved patient outcomes substantially in the early stages of bipolar disorder [19].

Inspired by experiences from the specialized Copenhagen Affective Disorder Clinic, and the King’s College/Institute of Psychiatry, London, the Mental Health Services, Capital Region of Denmark, decided to implement and test a new organization of outpatient treatment services for all patients with bipolar disorder who are not newly diagnosed, but in a more progressed state (secondary care facilities), in the Capital Region, a so-called Clinical Academic Group for bipolar disorder (the CAG Bipolar) starting from December 2019 [20]. Clinical Academic Groups (CAGs) bring together clinical services, research, education, and training to offer care and treatment that is based upon reliable evidence backed up by research (https://www.kcl.ac.uk/ioppn/depts/ps/about/cags/index.aspx). A major aim of the CAGs is to aid effective and rapid use of the latest research to improve the care and treatment provided.

### Smartphones as a new way to empower patients, treatment, and research

The rapid growth in access to and capabilities of digital health technologies presents a feasible route toward augmenting traditional mental healthcare and bridging the gap between the need for treatment and the capacity to deliver it. In addition, the rapid evolution and ubiquity of mobile networks have resulted in the increasing development of smartphone-based tools for remote and real-time self-monitoring [21, 22]. Smartphones provide a unique platform for fine-grained symptom monitoring and treatment. They are a convenient means of electronic self-assessment, allowing patients to record and review their data ubiquitously on-demand daily. The term “digital phenotyping” was introduced to mental health in 2015 [23] and has been defined as the “*moment-by-moment quantification of the individual-level human phenotype *in situ* using data from personal digital devices*” [24]. Digital phenotyping (both active (self-monitoring) and passive (sensor-based data)) refers to approaches in which personal data gathered from mobile devices and sensors is analyzed to provide health information on physiological functions or behavioral indicators [25, 26]. Digital phenotyping potentially uses smartphones to measure symptoms and behavior. These data can be seen as digital footprints or data traces arising as a by-product of interactions with technology. The rapid evolution and ubiquity of mobile technology have increased the development of tools for remote and real-time self-monitoring and treatment [27].

During the last decade, we have developed and tested a unique smartphone-based monitoring and treatment system, the Monsenso system for patients with bipolar disorder. The Monsenso system was initially developed by the IT University in Copenhagen in close collaboration with Copenhagen Affective Disorder Research Center (CADIC), Psychiatric Center Copenhagen, Mental Health Services, Capital Region of Denmark, involving a wide range of patients, clinicians, and researchers (in the EU funded MONARCA project [28, 29], the MONARCA II trial [30], and the RADMIS trial [31]). Using the Monsenso system for smartphone-based treatment of bipolar disorder has been shown to have a very high compliance rate (87–95%) and is considered useful by patients and clinicians helping patients to manage their course of illness [28–30]. Besides subjective monitoring, we have additionally shown that sensor-based data such as physical [32], social activity [33], and voice features [34, 35] reflect illness activity. The effects of the Monsenso smartphone system in patients with bipolar disorder have so far been investigated in three RCTs by the authors [29–31]. Overall, using the smartphone-based monitoring system resulted in increased quality of life, decreased perceived stress, and increased use of lithium without influencing the degree of depressive or manic symptoms. A recent systematic review and meta-analysis conducted by the International Society for Bipolar Disorders (ISBD) Big Data Task Force found no support that smartphone interventions may reduce the severity of depressive or manic symptoms in patients with bipolar disorder. However, the low number and high heterogeneity of studies supports the need for the use of more sensitive outcomes, such as psychiatric hospitalizations and mood instability [18].

### A window of opportunity

This is the first time that large-scale treatment with smartphones in clinical practice will be introduced and investigated as part of an outpatient psychiatric service in patients with bipolar disorder. Furthermore, it is the first time that mood instability is included as an outcome measure in an RCT investigating the effect of smartphone-based monitoring and treatment. The SmartBipolar trial will provide unique experiences of implementation of smartphone-based treatment for future trials and clinical practice in other psychiatric disease areas. A central aspect of the present SmartBipolar trial will be that patients with bipolar disorder are encouraged to conduct daily self-evaluations of symptoms. Firstly, this will empower the patients themselves (and their relatives) by increasing self-insight and self-treatment. Secondly, this will empower the clinicians by providing ongoing daily patient data increasing knowledge of the patient state and adherence to treatment between out-patient visits making early support and individualized treatment intervention possible. Finally, this study setup will facilitate research by providing continued and fine-grained daily patient-reported and automatically generated smartphone-based data on illness and treatment and thereby allowing for detailed and fine-grained identification of patterns.

### Hypotheses

In patients with bipolar disorder receiving specialized outpatient treatment for patients with bipolar disorders, using daily smartphone-based monitoring and treatment, *including* clinical feedback, improves mood instability and other clinically relevant outcomes (specified below) more than daily smartphone-based monitoring *without* clinical feedback or daily smartphone-based mood monitoring *only*.

### Objectives

The objectives are as follows: to investigate in a randomized controlled parallel-group trial whether (1) daily smartphone-based outpatient monitoring and treatment *including* clinical feedback versus (2) daily smartphone-based monitoring *without* clinical feedback or (3) daily smartphone-based mood monitoring *only* improves mood instability in patients with bipolar disorder and, furthermore, to investigate if daily smartphone-based monitoring, *including* clinical feedback, improves *questionnaire-based* quality of life, self-rated depressive symptoms, self-rated manic symptoms, perceived stress, and satisfaction with care, as well as *register-based* risk of psychiatric hospitalization and cumulated days of psychiatric admission as compared with daily smartphone-based monitoring *without* clinical feedback and daily smartphone-based mood monitoring *only* in patients with bipolar disorder. In addition, this is to investigate if daily smartphone-based monitoring, *including* clinical feedback, improves *smartphone-based* measures of stress, anxiety, irritability, activity, sleep duration, days with adherence to medication, days without mixed mood, and adherence to smartphone-based monitoring as compared with daily smartphone-based monitoring *without* clinical feedback in patients with bipolar disorder.

## Methods

The present trial protocol is reported according to the CONsolidated Standards Of Reporting Trials (CONSORT) statement and Standard Protocol Items: Recommendations for Interventional Trials (SPIRIT) [36–38] (Table 1 and Additional file 1).

**Table 1:** Schedule of enrolment, interventions, and assessments in the SmartBipolar randomized controlled trial according to the SPIRIT checklist

Study period		
	**Baseline**	**Post allocation**
**TIMEPOINT**	0 months	6 months
**Enrollment**		
Informed consent	X	
Allocation	X	
**Interventions**		
Daily smartphone-based monitoring and treatment *with* clinical feedback loop	X	X
Daily smartphone-based monitoring and treatment *without* clinical feedback loop	X	X
Daily smartphone-based mood monitoring only	X	X
**Assessments**		
Smartphone-based mood monitoring	Daily during the entire follow-up period	
Questionnaires^a^	X	X
Registry data^b^		X

^a^Quality of life according to WHO Quality of Life-BREF (WHOQOL-BREF); Self-rated depressive symptoms according to Major Depressive Inventory (MDI); Self-rated manic symptoms according to Altman Self Rating scale for Mania (ASRM); Perceived stress according to Cohen’s Perceived stress scale; Satisfaction with care according to Verona Satisfaction Scale-Affective Disorder (VSS-A)

^b^Number and duration of psychiatric hospitalization during follow-up, and use of medication during follow-up

The trial protocol describes a randomized controlled parallel-group superiority trial, the SmartBipolar trial, investigating the effect of smartphone-based monitoring and treatment in adult patients with bipolar disorder. The SmartBipolar trial is part of a larger CAG bipolar trial [20] and therefore has some of the same secondary and tertiary outcome measures.

### Trial design and study organization

The SmartBipolar trial is designed as a pragmatic randomized controlled parallel-group trial with a balanced allocation ratio (1:1:1) of adult patients with bipolar disorder stratified according to psychiatric center from where the patients receive outpatient treatment as part of the CAG Bipolar specialized outpatient treatment (Psychiatric Center Copenhagen, Psychiatric Center North Zealand, Psychiatric Center Amager, Psychiatric Center Glostrup, Psychiatric Center Ballerup in the Capital Region of Denmark). The patients are randomized to the SmartBipolar trial during a 6-month trial period (see description of intervention below).

**Fig. 1:**
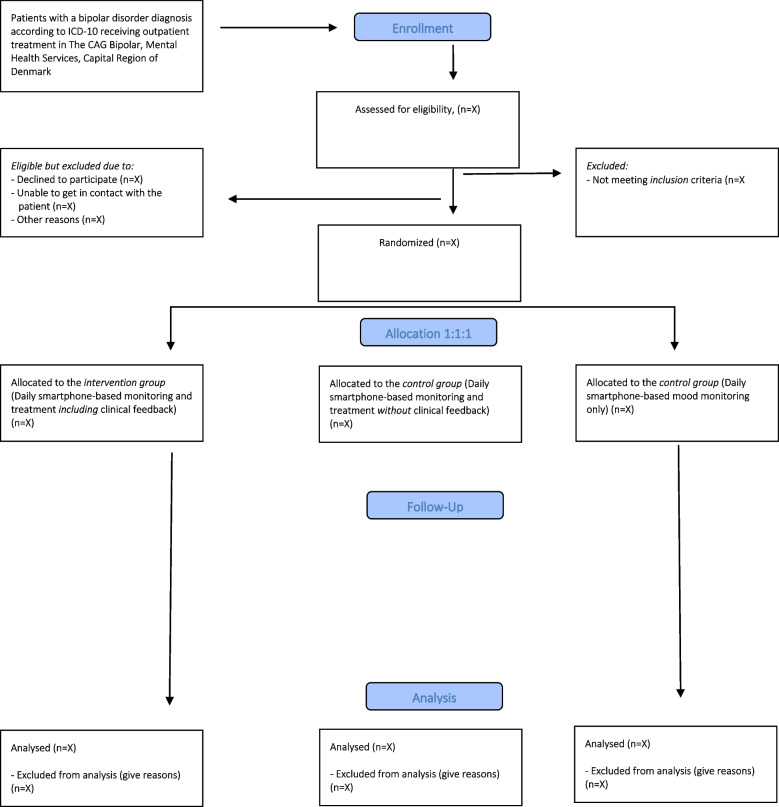
Flow diagram, the SmartBipolar randomized controlled trial

The included patients are randomized to either active use of the entire Monsenso system, including clinical feedback (the intervention group), or to the use of the entire Monsenso system without clinical feedback (control group 1) or to mood monitoring only (control group 2). Patients continue with treatment as usual during the trial period. The flow diagram of the SmartBipolar trial is presented in Fig. 1. The SmartBipolar trial is conducted at the psychiatric centers listed above. No changes in study design or methods have been made after trial commencement.

### Participants and settings

All patients with a bipolar disorder diagnosis according to the International Classification of Diseases, version 10 (ICD-10), receiving treatment in the CAG Bipolar outpatient clinics in the Mental Health Services, Capital Region of Denmark, are invited to participate in the trial. The staff consists of specialists in psychiatry and psychologists, as well as nurses, social workers, and recovery mentors with a specific interest and clinical experience and knowledge about the diagnosis and treatment of bipolar disorder. The physicians follow evidence-based psychopharmacological treatment as according to established guidelines [39], and treatment also encompasses group-based psychoeducation, coordinated targets to improve quality of life by increasing concordance between clinicians, patients, and relatives as well as continued ongoing supervision by a specialized clinical team [20].

The inclusion criteria are as follows: patients over the age of 18 years with a bipolar disorder diagnosis according to the International Classification of Diseases, version 10 (ICD-10), who are receiving treatment in the CAG Bipolar outpatient clinics in the Mental Health Services, Capital Region of Denmark, are eligible for inclusion. Thus, the patients are not newly diagnosed but receiving treatment for bipolar disorder during a more progressed state. Inclusion started in March 2021. The exclusion criteria are as follows: patients who do not own and use a smartphone and who lack Danish language skills are excluded. Patients meeting the inclusion criteria and having none of the exclusion criteria are invited to be enrolled in the SmartBipolar trial.

### Study procedure

All potential patients are invited to participate in the SmartBipolar trial by the staff from the CAG Bipolar outpatient clinics the Mental Health Services, Capital Region of Denmark. Since patients are recruited from the CAG bipolar clinics, all patients are diagnosed with bipolar disorder according to ICD-10 by highly trained clinicians with a specific expertise within bipolar disorder. Patients who accept to participate in the SmartBipolar trial are screened by trained clinicians to make sure they fulfil the criteria for participation. Following inclusion, the included patients are randomized separately according to psychiatric center (Psychiatric Center Copenhagen, Psychiatric Center North Zealand, Psychiatric Center Amager, Psychiatric Center Glostrup, Psychiatric Center Ballerup) to either the intervention group or one of the two control groups for a six-month trial period.

An overview of the data collection during the trial is presented in Table 1. Information regarding psychiatric hospitalization and duration of admissions, and the use of medication, will be obtained after completion of the RCT by linkage of the unique personal identification number (CPR), which is assigned to all 5.9 million persons living in Denmark with the Danish Psychiatric Central Register [40].

### The intervention group

The authors have developed and tested a unique smartphone-based monitoring and treatment system, the Monsenso system, for patients with bipolar disorder. The Monsenso solution was initially developed by the IT University in Copenhagen in close collaboration with the Copenhagen Affective Disorder Research Center (CADIC), Psychiatric Center Copenhagen, Mental Health Services, Capital Region of Denmark, and a wide range of patients, clinicians, and researchers (in the EU funded MONARCA project [28, 29], the MONARCA II trial [30], and the RADMIS trial [31]). The effects of the Monsenso smartphone system in patients with bipolar disorder have so far been investigated in three RCTs by the authors [29–31]. Overall, using the smartphone-based monitoring system resulted in increased quality of life, decreased perceived stress, and increased use of Lithium without influencing the degree of depressive or manic symptoms.

Following the MONARCA studies and the RADMIS trial, adjustments to the Monsenso system were made, which now provides the following main features:

- Offer patients and clinicians across the Mental Health Services in the Capital Region of Denmark a tool for digital monitoring and support of treatment as a common way to substitute the general use of paper-based week schemes and clinical questionnaires.
- Make psychoeducation, personal treatment plans, and cognitive behavioral therapy tools easily assessable via smartphone as self-help between clinical appointments.
- Collecting time series of data consisting of daily self-monitoring (mood, activity, sleep, irritability, mixed mood, cognitive problems, anxiety, alcohol consumption, stress, medicine consumption, menstruation, notes) and clinical questionnaires as well as sensor data from smartphone aiming to:
  - ◦ Provide an overview of clinical status, behavior, and adherence to and effect of medication and psychological therapy
  - ◦ Use data to intensity research in CAG bipolar clinics
  - ◦ Gain insight into psychopathology and treatment on sub-group levels (e.g., according to age and gender, bipolar subtype I or II)
  - ◦ Optimize ongoing work with algorithms to predict relapse and episodes

The patients included in the SmartBipolar trial are invited to conduct smartphone-based evaluations in the Monsenso system daily. Patients in the intervention group and the first control group are invited to conduct evaluations of symptoms daily. Patients in the second control group are invited to conduct daily monitoring of mood only. In the two control groups, a study nurse without interfering in any way with the patients’ treatment will continuously monitor the adherence to smartphone-based self-monitoring, and in the case of lacking self-monitoring, the patients will during the entire trial period be contacted and encouraged to fill in the data. Both the intervention group and the control groups are using their smartphones for the 6-month trial period. In the SmartBipolar trial, the Monsenso system is available for smartphones capable of collecting sensor-based data on measures of illness activity on various levels (e.g., different levels according to type of smartphone—versions of Android smartphones and iPhones).

#### Subjective (self-monitored) measures of illness activity in the intervention group

**Fig. 2:**
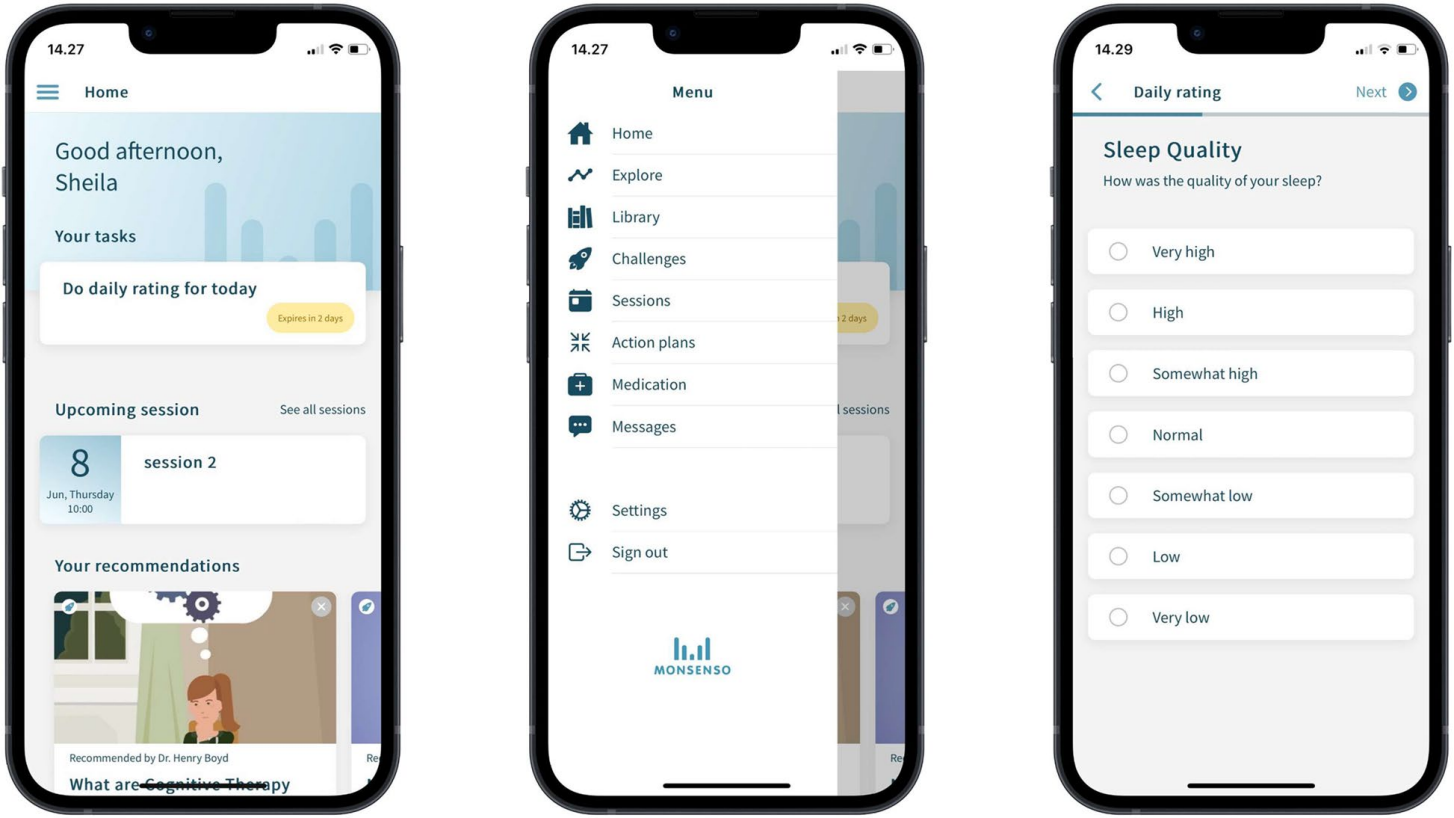
Screenshot of the smartphone-based self-assessment

**Fig. 3:**
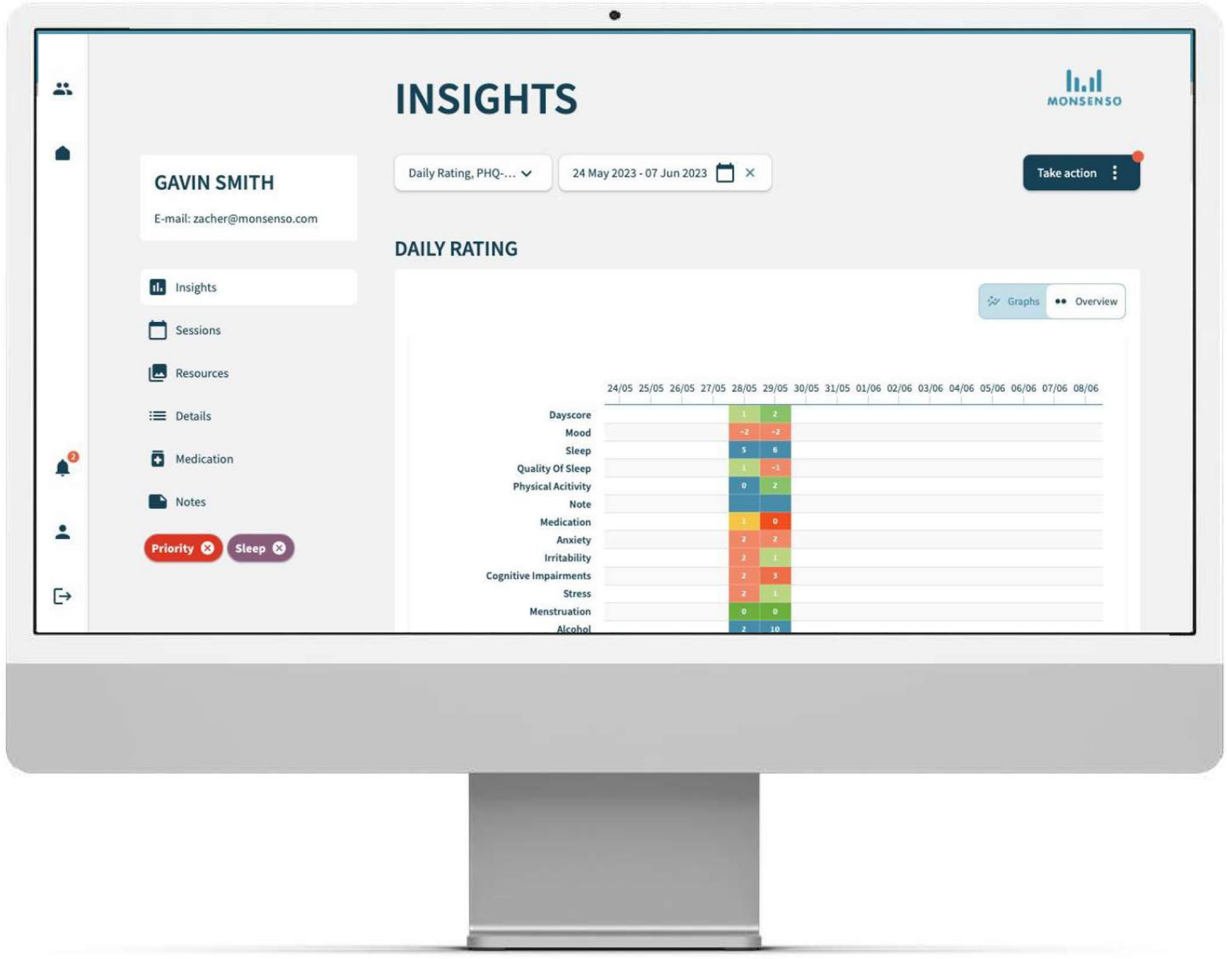
Screenshot of the web portal used by the clinicians for the feedback

The patients randomized to the intervention group are prompted by an alarm in the Monsenso system at a self-chosen time during the day (standard 7 pm but can be changed and also be de-activated by the patient) to evaluate subjective measures of illness activity daily. The following subjective (self-monitored) measures of illness activity are available for evaluation: mood (scored from depressive to manic on a scale from − 3, − 2, − 1, − 0.5, 0, + 0.5, + 1, + 2, + 3), sleep duration (number of hours slept per night, measured in half-hour intervals), medicine intake (taken as prescribed/taken with changes/not taken), activity level (scored from very low to very high on a scale from − 3, − 2, − 1, 0, + 1, + 2, + 3), mixed mood (yes/no), irritability (scored from not present, present to some degree, present, present most of the time on a scale from 0, 1, 2, 3, 4), anxiety (scored from not present, present to some degree, present, present most of the time on a scale from 0, 1, 2, 3, 4), cognitive problems (scored from not present, present to some degree, present, present most of the time on a scale from 0, 1, 2, 3, 4), alcohol consumption (number of units consumed per day, 0 to + 10 scale), stress (scored from not present, present to some degree, present, present most of the time on a scale from 0, 1, 2, 3, 4), menstruation for women (yes/no), a number (unlimited) of personal parameters (created by the patients themselves), and a free-text note. A patient can edit their responses in a self-assessment until midnight and the latest given response will be saved and shown in the web portal. If the patients wish to change their self-reports, they can enter a second evaluation in addition to the initial one, and both of the subjective evaluations are then visible for the patient and the healthcare provider when logging on to in the Monsenso system. If the patients forget to evaluate the subjective measures, they can evaluate retrospectively for up to two days. Pictures of the self-reported mood monitoring are presented in Figs. 2 and 3.

#### Smartphone-based cognitive behavioral therapy (CBT) modules

Within the Monsenso system, the following CBT modules are available: psychoeducation, behavioral activation, cognitive restructuring, and rumination-focused CBT [41]. The psychoeducation and mood-monitoring system include strategies for detecting and intervening with early signs of relapse. Patients are encouraged to use the module in an individualized and sequential way according to their condition, affective state, and insight and following guidance from the study nurse if wanted. The behavioral activation module includes activity monitoring and activity scheduling and focus on regulation of sleep and daily routines. The cognitive restructuring includes simple techniques to help patients to identify and modify dysfunctional automatic thoughts. A main purpose of the smartphone-based CBT modules was to help patients to reduce depressive ruminations. Therefore, a specific rumination-focused CBT strategy was included.

#### The clinical feedback

By offering all patients with bipolar disorder and clinicians to use the system, a unique world class data set is made, which can be used to optimize treatment to the benefit of patients and clinicians. Nevertheless, it is unclear and has never been investigated how smartphone-based treatment is best optimized—with or without a clinical feedback loop. The clinical feed-back loop includes (1) that clinicians continuously monitor self-reported patient smartphone data making early intervention possible when data suggest deterioration; (2) actively support and help patients employing the Monsenso tools including psychoeducation, personal treatment plans, and cognitive behavioral therapy tools; (3) integration of the Monsenso system in group-based psychoeducation courses in the CAG bipolar clinics; and (4) meetings with the staff from the entire CAG bipolar clinic on a weekly basis where Monsenso registrations are monitored and discussed in collaboration (board meetings).

It is possible that patients will be able to use the Monsenso system entirely as a self-help tool in addition to treatment in the CAG bipolar clinics (see first control group below).

### The control group

#### The first control group

Patients allocated to the first control group are invited to conduct daily smartphone-based monitoring *without* a clinical feedback loop. Furthermore, they will have full access to the smartphone-based CBT modules as described above.

#### The second control group

Patients allocated to the second control group are invited to conduct daily smartphone-based mood monitoring only.

##### Sensor-based data

Smartphones capable of collecting sensor-based data on measures of illness activity regardless of randomization group (intervention group or control group) collects as many sensor data daily during the trial period as possible based on patient given consent (iOS and Android: Battery level, Step count, Activity, Geolocation. Android: Light and screen state (on, off)). Thus, we will be able to investigate the use of sensor-based data for prediction analyses etc.

All included patients continue with their treatment at the CAG bipolar clinic. After the end of the SmartBipolar trial, patients will be offered the full version of the Monsenso system including clinical feedback.

### Assessments

Since the SmartBipolar trial is a pragmatic trial and patients are recruited from the CAG bipolar clinics, all patients are diagnosed with bipolar disorder by highly trained clinicians with specific expertise within bipolar disorder. All outcomes, apart from registry-based measures, which are based on public registry-based data blinded for intervention, are based on patient evaluations (questionnaires and smartphone-based evaluations) and therefore assessed without blinding to the intervention. The patients are, regardless of randomization group, enrolled for a 6-month trial period and invited to fill out questionnaires two times during follow-up—at baseline and after 6 months (Table 1). Data collection of basic sociodemographic, clinical data, and outcome measures (besides the primary and registry-based data) including questionnaires will be handled electronically via the Redcap database in accordance with the standard procedure in the Capital Region of Denmark.

### Outcomes

#### Primary outcomes

- Mood instability (based on daily smartphone-based measures of mood)

#### Secondary outcomes

- Patient-based questionnaires collected at baseline and after six months using the following questionnaires: quality of life according to WHO Quality of Life-BREF (WHOQoL) [42]; self-rated depressive according to the Major Depressive Inventory (MDI) [43]; self-rated manic symptoms according to the Altman Self-rating Scale for Mania [44]; perceived stress according to Cohen’s Perceived stress scale (PSS) [45]; satisfaction with care according to scores on the Verona Satisfaction Scale-Affective Disorder (VSS-A) [46]. The validity of each questionnaire is available from the references. Patients will be contacted by the study nurse in case on non-response of the questionnaires. Furthermore, we will collect information on adherence to the Danish national guidelines of medical treatment of bipolar disorder according to use of the three main maintenance mood stabilizers for bipolar disorder: lithium, lamotrigine or quetiapine [39]. Risk of psychiatric hospitalization and cumulated number and duration of psychiatric hospitalization according to data from the population-based Danish Psychiatric Central Research Register [40].

#### Tertiary outcomes (based on daily smartphone-based measures)

The following outcomes will be compared between the intervention group and the first control group.

- Mean stress (per month during the trial)
- Mean anxiety (per month during the trial)
- Mean irritability (per month during the trial)
- Mean activity (per month during the trial)
- Mean sleep duration (per month during the trial)
- Percentage of days with mixed mood
- Percentage of days with adherence to medication during the trial period
- Adherence to smartphone-based self-monitoring during the trial period

No changes in outcome measures have been made after trial commencement. All patients will be contacted by the study nurse during the study to ensure retention and complete follow-up.

### Statistical power and sample size calculation

The statistical power and sample size were calculated using http://powerandsamplesize.com.

In the SmartBipolar trial with a follow-up period of 6 months, 1/3 will be randomized to daily smartphone-based monitoring and treatment, *including* a clinical feedback loop (intervention group). 1/3 will be randomized to daily smartphone-based monitoring, *without* a clinical feedback loop (control group), and 1/3 will be randomized to daily smartphone-based mood monitoring only (control group).

#### Mood instability

For each patient, a mood instability measure based on smartphone-based daily mood evaluations will be estimated for each day and aggregated in accordance with prior definitions by applying the root mean square successive difference (rMSSD) method [13, 14, 47]. The daily mood instability measures, reflecting the extent to which a daily and the previous day’s scores of self-monitored mood differed from one another during follow-up, were computed as the squared successive difference (SSD) of the reported values. Consequently, a daily mood instability measure could only be computed when consecutive daily values are present. The resulting SSD values will be aggregated for each patient as the root mean square successive difference (rMSSD), taking the square root of the mean of the SSD values [9, 11, 47, 48]. The instability measures will be computed following the original definition of the RMSSD score. Thus, differences between successive differences in mood are calculated, and then the values are squared, and the results are averaged before the square root of the total is obtained. The differences will be squared such that positive and negative differences do not cancel out when the values are aggregated by computing the mean. Squaring the values also puts more weight on larger differences. Finally, the square root of the mean puts the aggregated value back on the original scale. rMSSD is one of a few time-domain tools used to assess variability, the successive differences being neighboring mood scores. We decided to apply the RMSSD method following previous work. Thus, the mood instability measure do not reflect the severity of self-monitored data but rather the day-to-day changes by a statistical measure of a response to a question.

The primary outcome is mood instability between the intervention group and the two control groups—calculated as described above. Analyses will be done using intention-to-treat analyses. According to recent analyses, [15] mood instability in patients with bipolar disorder calculated using the definition above has a mean of 0.80 (SD 1.93). If mood instability decreases 0.2 units (SD 0.53) on the mood instability scale for trial arm I (the intervention group) versus trial arm II (the first control group) and trial arm I versus trial arm III (the second control group), respectively, a drop-out rate of 10% during the 6-month trial period (in accordance with the drop-out in our prior 6 months smartphone RCT) [30], a two-sided risk of type 1 error, *α* of 0.025 (since we will conduct two analyses between three groups with 0.05 level of significance in each analyses), a type 2 error risk, *β*, and a statistical power of 80%, the sample size (*N*) is calculated as *N* = 183. Thus, we need to allocate 67 patients in each of the tree arms of the trial and to randomize a total of 201 patients in the SmartBipolar trial.

### Randomization

#### Sequence generation

The company randomize.net is used for the randomization (http://randomize.net). The randomization is conducted by the staff at each center using an online procedure with stratification according to the psychiatric center. Each center can log on to a webpage by using a secure code and will conduct the randomization on-site. Patients included in the trial are randomized with a balanced allocation ratio of 1:1:1 to (1) daily smartphone-based monitoring and treatment *including* a clinical feedback loop (intervention group) or (2) daily smartphone-based monitoring *without* a clinical feedback loop (control group) or (3) daily smartphone-based mood monitoring only (control group) (Table 1). Block sizes are (unavailable to those who enroll participants or assign interventions) used to help preserve unpredictability [49, 50]. The study uses a stratified design, where patients are stratified according to the CAG bipolar clinic.

The statistical analyses will be adjusted for the stratification variable and age and sex as possible prognostic variables. Furthermore, in analyses on continuous variables, potential differences in baseline scores on the outcome in question will be included as a potential confounder. If there is no statistically significant main effects of age and sex, these variables will be excluded from the final statistical analyses.

### Blinding

Owing to the type of intervention in the SmartBipolar RCT, the patient, the patient’s healthcare provider, and the researchers are aware of the allocated randomization group. However, data on psychiatric hospitalization can be assessed, blinded to the intervention status, by the researchers. The researchers are responsible for data entry, data analyses, interpretation of analyses, and writing of papers are kept blinded to allocation during the handling of data.

### Statistical methods

Data from all randomized patients is collected until dropout or the end of the trial period. The analysis will be carried out with an intention-to-treat (ITT) approach. Concerning the primary, secondary, and tertiary outcomes, analyses will be done employing linear mixed-effects regression models with a random intercept for each participant. Differences in outcomes between the intervention group and the control groups will be analyzed, firstly in an unadjusted model (except for potential differences in baseline values of the outcome variable in analyses) and then in models adjusted for the stratification variable CAG bipolar clinic and for age and sex as possible prognostic variables. If there is no statistically significant main effects of age and sex, these variables will be excluded from the final analyses. Multiple regression models will be conducted investigating differences in the proportion of psychiatric hospitalizations and the cumulated duration of all psychiatric hospitalizations during the sex-month trial period. Models will be conducted as unadjusted and adjusted for CAG bipolar clinic (stratification variable), age, and sex. Missing data will be handled as missing at random.

For all statistical analyses, the statistical threshold for significance is *p* ≤ 0.05 (two-tailed). Data will be managed by LVK and MFJ and entered using RedCap®. All analyses will be done using SPSS, version 22.0 (IBM, New York, NY, USA), and STATA version 13 (StataCorp LP, College Station, TX, USA).

Data about potential and enrolled participants will be stored in RedCap® and on secure servers at Monsenso during and after the trial. Data collected during the course of the trial will be kept strictly confidential and only accessed by members of the trial team. Participants be allocated an individual trial identification number. Participant’s details will be stored in a secure database. Only researcher from the trial team will access the data set. Potentially, we will share anonymized trial data with other researchers to enable international prospective meta-analyses. This will be applied for separately.

### Ethics, data permission, registration, and dissemination

Ethical permission for the SmartBipolar trial was obtained from the Regional Ethics Committee in The Capital Region of Denmark and the data agency, Capital Region of Copenhagen (H-19067248, (P-2019–809)). The law on handling of personal data will be respected. The patients’ healthcare journals will be read to confirm information regarding the patients’ clinical history. The trial was registered at ClinicalTrials.gov as NCT0423042 January 2020. All positive, neutral, and negative findings of the trial will be published according to the CONSORT guidelines [36]. All potential participants are invited to receive information about the SmartBipolar trial on an individual basis by their mental healthcare professional where the information is given in a quiet and undisturbed room. All information is presented in both written and verbal form and participants can bring a friend or relative to the introduction conversation. Participants are informed that participation is voluntary and that consent can be withdrawn at any time during the trial without this having any consequences for future treatment possibilities. All included patients sign a consent form and get a copy of this and their rights as a participant in a clinical trial. Patients do not receive economic compensation for participating in the SmartBipolar trial. This trial does not involve collecting biological specimens for storage. Results will be published in peer-reviewed academic journals, presented at scientific meetings, and disseminated to patient organizations and media outlets. The coordinating center and trial steering committee consist of Prof. Lars Vedel Kessing, Ass. Prof. Maria Faurholt-Jepsen, Malene Schwarz Dyreholt, and Natacha Blauenfeldt Køster. Maria Faurholt-Jepsen, Malene Schwarz Dyreholt, and Natacha Blauenfeldt Køster provide day to day support for the trial. Lars Vedel Kessing, Maria Faurholt-Jepsen, Malene Schwarz Dyreholt,, and Natacha Blauenfeldt Køster are responsible for all aspects of local organization including identifying potential recruits and taking consent. Lars Vedel Kessing and Maria Faurholt-Jepsen will be auditing trial conduct four times annually. Lars Vedel Kessing, Maria Faurholt-Jepsen, and Nanna Iversen are supervising the trial and will meet four times annually. There are no stakeholder and public involvement groups. Interim analyses will not be performed. All potential positive, negative, and neutral effect will be reported. Only the researchers will have access to the final dataset. All data including the smartphone-based data are owned by the researchers. Any data required to support the protocol can be supplied on request. The authors wish not to share smartphone-based data since they can easily be sensitive. Statistical codes can be shared upon request.

There is no data monitoring committee since this is a low-risk intervention being trialed. There is no evidence that suggests that serious adverse events would be anticipated. Potential minor adverse event may be anticipated. For this reason, we monitor worsening in severity of depressive and manic symptoms.

## Discussion

Psychiatric disorders represent a major disease burden worldwide with a significant impact on the quality of life, socioeconomic functioning, and life expectancy [51]. At the same time, there is a gap between the need for treatment and the number of patients receiving treatment. The scarcity of treatment resources leads to long waiting times, and stigma surrounding mental health issues may prevent individuals from seeking help or receiving appropriate treatment [52].

In 2011, the World Health Organization stated that “the use of mobile and wireless technologies to support the achievement of health objectives (mHealth) has the potential to transform the face of health service delivery across the globe” [53]. The number of smartphone mobile network subscribers exceeded 6 billion people in 2022 [54]. Digital treatment strategies, including smartphone-based interventions, have emerged as promising approaches to address challenges in mental health services [21, 22]. Smartphone-based treatment interventions are easy to use by patients in various geographical locations and provide support between outpatient’s appointments. Furthermore, smartphone-based treatment interventions can be accessed at the patient’s convenience and offer flexibility in engaging with their treatment allowing them to follow their own pace in comfortable settings. Using smartphone-based treatment interventions allows for personalized approaches tailoring and addressing the treatment to the patient’s specific needs and preferences. Also, the personalization and tailoring to specific needs and preferences may enhance user engagement, motivation, empowerment, and illness insight [21].Combining outpatient treatment and smartphone-based monitoring, including clinical feedback, may lead to better treatment and adherence and optimize treatment outcomes by providing continuous support, self-monitoring, and psychoeducation between clinical appointments [55]. COVID-19 and the transition of increased digital treatment strategies have shown us the potential for digital innovations to improve access and quality of care in mental health.

More than 50% of patients with bipolar disorder experience between episodes subsyndromal mood swings daily [8–10]. This mood variation is reflected as mood instability reflecting core psychopathology in bipolar disorder [9–12]. Furthermore, mood instability has been associated with increased perceived stress, decreased quality of life, and functioning as well as increased risk of relapse and hospitalization [9, 12–14]. In addition, mood instability has been suggested to be associated with biological measures of stress-hormone dynamics [16]. Due to the increased focus on the impact of mood instability, mood instability has been suggested as an independent treatment target and as a more sensitive measure of outcome in randomized controlled trials (RCTs) than for example relapse or recurrence of affective episodes [7, 8, 11, 17].

The SmartBipolar trial is the first RCT investigating the effects of smartphone-based monitoring and treatment including clinical feedback on mood instability in patients with bipolar disorder. As it unknown and never has been investigated whether smartphone-based monitoring and treatment including clinical feedback is better than smartphone-based monitoring and treatment or smartphone-based mood monitoring only in specialized outpatient clinics for patients with bipolar disorder, it is highly important to compare the efficiency of these interventions in an RCT. Even if no difference is found on the primary outcome measures, effects in relation to the secondary and tertiary outcome measures are important. The SmartBipolar trial is designed with sufficient statistical power to investigate differences concerning secondary and tertiary outcome measures.

### Advantages

The SmartBipolar trial uses a pragmatic design with few exclusion criteria, and the results of the trial will be generalizable to patients with progressed bipolar disorder in general (not newly diagnosed patients with bipolar disorder), that is, real-world patients with any kind of comorbidity, and have clinical relevance. The patients are recruited during their outpatient treatment and have need for continued treatment, and the Monsenso system is developed to support this treatment. Furthermore, it is favorable that the primary outcome measure is mood instability as this implies several advantages: mood instability has internal validity as a real-life measure for patients and high external validity as it reflects patients’ illness severity and functioning [13, 14, 16]. Mood instability appears to be a more sensitive measure of outcome in RCTs compared to more conventional outcomes focusing on relapse or recurrence of affective episodes. This is because, unlike traditional clinician rating scales that are labor intensive and capture brief periods of time, mood instability can be captured daily by self-report for long time periods (intermittently up to years) with high adherence and low cost from a large sample of individuals thus having the potential to increase statistical power and sensitivity to detect effects in RCTs [7]. In addition, instability of mood can be estimated using the root mean square of successive differences, a widely used measure of instability [11].

The modern healthcare sector is undergoing a profound transformation boosted by the ongoing adoption of digital interventions working towards the mergence of P4 healthcare—predictive, preventive, personalized, and participatory, that is working with shifting the focus from reactive and generalized treatment to proactive and individualized strategies to improve patient outcomes and overall wellbeing. The findings from the SmartBipolar trial will provide additional evidence to this development.

### Limitations

The SmartBipolar RCT is designed to investigate the effect of the entire smartphone-based monitoring including a clinical feedback loop in a specialized outpatient clinic for patients with bipolar disorder. Thus, we will not be able to distinguish the effects of the individual components of the intervention. Furthermore, besides the outcome measures of hospitalization, the outcome measures are unblinded as patients are aware of their allocation status.

### Generalization

The results of this RCT can be generalized to patients with bipolar disorder in general (not newly diagnosed patients with bipolar disorder), that is, real-world patients with any kind of comorbidity.

### Perspectives

The learning potential of the SmartBipolar trial is high due to its novelty, originality, and pragmatic design. The SmartBipolar trial is the first to investigate the effects of add-on smartphone-based treatment in mood instability for patients with bipolar disorder. The study findings will directly be implemented in the CAG bipolar outpatient clinics, potentially in a revised version according to the study findings. Moreover, findings will have great influence on the area of implementation of IT solutions in the mental health services in Denmark and internationally.

## Trial status

The trial started in March 2021 and has currently included 150 patients. The trial is ongoing. Recruitment began in March 2021. The estimated time for completed recruitment is March 2024. The present protocol is version 1, date: June 28, 2023.

### Supplementary Information

**Additional file 1. **SPIRIT 2013 Checklist.

## Abbreviations

RCT: Randomized controlled trial
The CAG Bipolar: The Clinical Academic Group for Bipolar disorder
The SmartBipolar: The smartphone-based treatment RCT for bipolar disorder
WHOQOL-BREF: The WHO Quality of Life-BREF
MDI: The Major Depressive Inventory
ASRM: The Altman Self-Rating Scale for Mania
PSS: The Perceived Stress Scale
VSS-A: The Verona Satisfaction Scale-Affective Disorder
ITT: Intention-to-treat
